# Impact of FO Operating Pressure and Membrane Tensile Strength on Draw-Channel Geometry and Resulting Hydrodynamics

**DOI:** 10.3390/membranes10050111

**Published:** 2020-05-25

**Authors:** Alexander J. Charlton, Boyue Lian, Gaetan Blandin, Greg Leslie, Pierre Le-Clech

**Affiliations:** 1 UNESCO Centre for Membrane Science and Technology, School of Chemical Engineering, University of New South Wales (UNSW), Sydney, NSW 2052, Australia; alexander.charlton@unsw.edu.au (A.J.C.); b.lian@unsw.edu.au (B.L.); g.leslie@unsw.edu.au (G.L.); 2 Eurecat, Centre Tecnològic de Catalunya, Water, Air and Soil Unit, 08005 Manresa, Spain; gaetan.blandin@lequia.udg.cat; 3 Institut Européen des Membranes, UMR5635, 34090 Montpellier, France

**Keywords:** forward osmosis, computational fluid dynamics (CFD), spacers, draw channel, pressure assisted osmosis

## Abstract

In an effort to improve performances of forward osmosis (FO) systems, several innovative draw spacers have been proposed. However, the small pressure generally applied on the feed side of the process is expected to result in the membrane bending towards the draw side, and in the gradual occlusion of the channel. This phenomenon potentially presents detrimental effects on process performance, including pressure drop and external concentration polarization (ECP) in the draw channel. A flat sheet FO system with a dot-spacer draw channel geometry was characterized to determine the degree of draw channel occlusion resulting from feed pressurization, and the resulting implications on flow performance. First, tensile testing was performed on the FO membrane to derive a Young’s modulus, used to assess the membrane stretching, and the resulting draw channel characteristics under a range of moderate feed pressures. Membrane apex reached up to 67% of the membrane channel height when transmembrane pressure (TMP) of 1.4 bar was applied. The new FO channels considerations were then processed by computational fluid dynamics model (computational fluid dynamics (CFD) by ANSYS Fluent v19.1) and validated against previously obtained experimental data. Further simulations were conducted to better assess velocity profiles, Reynolds number and shear rate. Reynolds number on the membrane surface (draw side) increased by 20% and shear rate increased by 90% when occlusion changed from 0 to 70%, impacting concentration polarisation (CP) on the membrane surface and therefore FO performance. This paper shows that FO draw channel occlusion is expected to have a significant impact on fluid hydrodynamics when the membrane is not appropriately supported in the draw side.

## 1. Introduction

Forward osmosis (FO) membrane processes have undergone significant development in both the research field and the commercial production of modules. The interest in FO was initially drawn by the low operating pressures and implicated economic benefits when compared to conventional reverse osmosis (RO) membrane processes [1]. FO has also been investigated as an alternative for conventional wastewater treatment, most recently concerning hybrid systems that incorporate both RO and FO by utilizing an osmotic dilution step preceding the conventional RO step to reduce overall membrane fouling [2,3,4,5]. The viability of FO has been researched in the treatment of textile industries wastewater, oil and gas well-fracturing, osmotic concentration of liquid foods, and other novel applications, yet with limited current industrial implementation and largely lab-scale studies [6,7,8]. However, specialized applications such as utilizing fertilizer as a draw solution or brine dilution show continued industrial and commercial interest [9].

Limitations of the process include the identification of a suitable draw solution, issues with reverse solute flux, internal concentration polarisation (ICP), external concentration polarisation (ECP) and finally pressure considerations within the module [1,10,11]. Concentration Polarisation (CP) particularly affects FO processes due to the significance of osmotic pressure as a driving force. ICP is specific to FO membranes, occurring within the porous support layer of the membrane. This concentration gradient causes an increase in solute concentration at the active-support interface, reducing the effective osmotic pressure difference across the membrane active layer and thus, the driving force resulting in lower fluxes [12]. In efforts to reduce the adverse effects of ICP on FO processes, membranes with thinner support layers haven been investigated [13]. In FO processes, mass transfer is important on both sides of the membrane, and as such ECP also occurs on the draw side in the form of dilutive ECP, which lowers the flux of the process and is further hypothesized to affect ICP in the support layer [14,15]. In particular, ECP has been found to occur in a significant degree on the draw-side to influence mass transfer and overall FO performance [16]. Overall, the effects of ICP and ECP still hinder the viable application of FO in an industrial context with fluxes generally too low for economical operation.

For FO successful implementation, it is also critical to consider that the process operates by the circulation of feed and draw solutions, practically implemented by pumping and applying suitable pressure, as indicated in experimental studies conducted between 0 and 3 bar of feed pressure [10,17,18]. During operation, the pressure in the feed-side is required to be higher than the draw-side so as not to oppose the osmotic gradient. This difference in pressure across the membrane, or transmembrane pressure (TMP), is the general operating for most membrane processes and is an assistive driving force in FO processes, in the form of AFO (assisted forward osmosis) [19]. TMP in FO processes has been hypothesized to cause a draw channel contraction in spiral-wound elements, to result in pressure drops along the draw-side of the membrane [17]. A relationship between this draw channel contraction and increased overall concentration polarisation (CP) has been established by utilizing expected pure water flux as a baseline, demonstrating the need for mechanical support to improve performance [20]. Spacers are mechanical supports between membrane layers used in modules to allow fluid flow in an open channel and promote mixing to reduce fouling and transport rejected substances from the membrane surface [21]. However, spacer designs need to balance appropriate mixing with low pressure drops for economical operation of the modules, leading to research and implementation of a wide range of spacer designs to optimize this tradeoff [22]. Novel spacer designs in FO do not need the high mechanical support of RO spacers, due to the lower operating pressures, resulting in the use of a ‘dot-spacer’ design specifically for FO draw-side operation [23]. With respect to spacer design and related pressure drops, initial computational fluid dynamics (CFD) investigation has been conducted to assess the effects of inlet pressure and cross-flow velocity (CFV) on the pressure loss by the fluid, confirming a change in draw channel height corresponds with pressure drop [10]. Plate-and-frame (PF) spacer design on the draw-side remains relatively unexplored, and current industrial design allows for membrane deformation under pressure, resulting in a potentially significant pressure drop. Further work towards a more accurate CFD model spacer design characterisation and assessment of novel spacer configurations is expected to improve draw-side spacers and overall FO module design and operation [24].

The tensile strength of membranes provides an indicator as to the operating pressure they can withstand and is highly dependent on the support structure of the membrane [25]. However, tensile strength in FO applications can be balanced against flux, as a higher tensile strength is correlated to lower flux caused by the resistance of thicker mechanical support structures [26,27]. The relationship between higher flux and thinner membranes leads the industry to strive for membranes as thin as mechanically possible to achieve economically high fluxes, without regard for the behaviour of membranes in operation under significant TMP. The tensile strength of FO membranes has been linked to deformation under higher feed pressure conditions, yet the direct relationship between the tensile characteristics and level of deformation has yet to be assessed [20,28].

The direct relationship between tensile strength and membrane deformation under pressure can provide insight into the draw channel occlusion in FO processes, and characterisation of the hydrodynamic effects has left a gap in the literature. Specifically, the level of expected occlusion under pressure, as well as flow profile effects by channel occlusion leave an unexplored set of parameters that could potentially allow for more finely tuned flux performance in real-world applications, increasing the viability of commercial FO. This paper, therefore, aims to assess the effect of membrane occlusion into the draw channel of FO processes, specifically plate-and-frame, and determine the subsequent effects on the fluid flow profile, pressure loss, and other performance indicators such as salt rejection.

## 2. Materials and Methods

### 2.1. CFD Modelling of Membrane Processes

**Figure 1:**
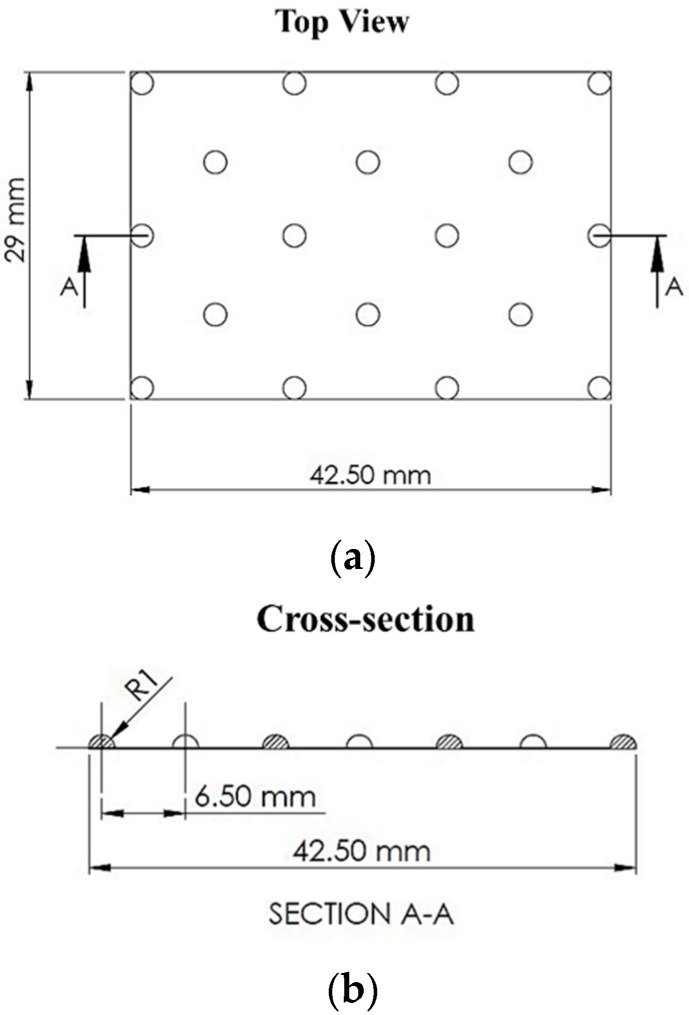
Plate and frame draw spacer used in experimental tests and ANSYS Fluent v19.1 analysis: (**a**) geometric view cut-out and (**b**) cross-section.

CFD modelling of FO processes cell is based on the method initially developed in [10], with modifications for further geometry and design parameter of the draw channel. All CFD simulations were performed using ^®^ANSYS Fluent v19.1. The 3D domains detail an outline dot spacer to represent the spacer from an industrial plate and frame module. Tetra mesh was employed for each fluid domain, with an average size of 0.2 mm. However, owing to file size limitations in ANSYS, only a portion of each spacer is represented as a segment of the total channel (300 × 400 mm) (Figure 1).

A no-slip wall condition was utilised to estimate the continuous flow between fluid and spacer, and the two sides of the membrane will be considered symmetrical for the purpose of simplification. All simulations will be run using the Semi-Implicit Method for Pressure Linked Equations (SIMPLE) algorithm for pressure-velocity coupling and First Order Upwind (FOU) algorithm for the discretization of the conservation equations. The draw channel was simulated in ANSYS as a single segment 65 mm in width to improve computational times, as the flow profile does not change across the module with respect to width, and pressure drop is linear.

### 2.2. Application of Tensile Membrane Characterisation into ANSYS Model

The tensile strength (MPa) of membranes was characterized using an INSTRON5565 (Instron, Canton, MA, USA). The membranes were cut into 20 × 5 mm sections [29] and tested with a ramp force increasing at a rate of 0.250 N/min to 180,000N [26].

The Young’s modulus (E) was obtained from a stress-strain curve of the membrane, based on the initial elastic region of the data. The modulus is a relationship between the stress applied and subsequent change in length Equation (1):

(1) $$E=\frac{\sigma }{\varepsilon }$$

where *E* is the Young’s Modulus, σ is the uniaxial stress, and ε is the strain.

Hook’s law was then used as means of relating the change in length to a force, where the law is a well-established relationship to be used as a basis for membrane elongation in Equation (2).

(2) $$∆L=\frac{F}{k}$$

where *F* is the force applied (as a TMP) and *k* is a proportionality constant.

For the case of uniaxial stress Hook’s law can be expanded with respect to *k*, accounting for the area to which the force is applied and the Young’s modulus of the material. This can be used to calculate a total elongation at a TMP across the membrane during FO operation.

(3) $$∆L=\frac{1}{E}\frac{F}{A}L_{0}$$

where *A* is the area to which the force is applied and $L_{0}$ is the original length.

Once the change in length of the membrane was calculated, the data can be used in a model that matches the ellipsoidal shape from the dot-spacer supports in the draw channel, used as a model of the geometry to be applied into ANSYS for further fluid flow profile characterisation, Equation (4).

(4) $$P_{ellipse}=2a\pi (1-\sum_{i=1}^{\infty }\frac{\left(2i\right){!}^{2}}{{\left(2\cdot i!\right)}^{4}}\cdot \frac{e^{2i}}{2i-1})$$

where P is the perimeter of the ellipse, and *a* is the major axis radius. The apex was then calculated using the minor axis of the ellipse plus twice the height of the apex along the dot spacer to simulate the slight ‘sag’ of the membrane against the spacer in the module. This new geometry was then used to inform the design of ANSYS geometry to determine fluid flow characteristics.

### 2.3. Wall Shear Rate

The shear rate on a wall is a tangentially exerted force; in the case of membrane processes, this is exerted onto the membrane surface. Mass transport away from the membrane surface on the draw side and into the bulk fluid flow is a key factor of FO process membrane performance. Increasing the shear rate on a membrane surface in an FO process is known to increase flux as decrease fouling and ECP. Wall shear rate has been reported in the literature to assess the hydrodynamics of FO channels in the form of numerical simulations [30]. ANSYS was used first to calculate an average shear rate value on the membrane surface and subsequent visualization utilizing a contour map of the shear rate throughout the chamber geometry. Equations (5) and (6) describe how ANSYS calculates shear rate of the bulk fluid and at surfaces.

(5) $$S_{ij}=\frac{1}{2}\left[\frac{\delta U_{i}}{\delta X_{i}}+\frac{\delta U_{j}}{\delta X_{j}}\right]$$

(6) $$\mathrm{Shear}\mathrm{Rate}={\left[\frac{\delta U_{i}}{\delta X_{i}}\right]}^{\frac{1}{2}}$$

where $S_{ij}$ is the rate tensor, $U_{i}$ is velocity and $X_{i}$ is the spatial coordinate.

### 2.4. Reynolds Number Analysis

Reynolds number was analyzed and compared to assess the impact of different channel geometries on flow behaviours related to mixing and turbulence. Reynolds number has been reported in the CFD assessment of membrane processes previously in narrow spacer-filled channels [30]. Reynolds number is extracted to indicate the potential improvement in flux outcomes in the draw-side channel, as reported in the literature [31]. While the Reynolds number can be found detailed in the literature, a summary of the factors is given in Equation (7).

(7) Re=ρvdueff

where ρ is density, d is cell volume, u is velocity magnitude and $u_{\mathrm{eff}}$ is the effective fluid viscosity.

### 2.5. Experimental Setup

The experimental data used in this study to validate the novel CFD analysis was obtained in a previous study and the key operating conditions of the experiments are as follows [10]. The membrane used was supplied by Porifera Incorporated (San Leandro, CA, USA) (Model no. PF-20), which used a diamond-type polypropylene feed spacer and printed dot-spacer for the draw side. The total membrane area is 1 $m^{2}$, tested at pressures from 0–4 bar. Feed solution was pumped using a high pressure pump (Procon Systems Inc., Canada); draw solutions were pumped using a peristaltic pump (Masterflex, USA). Further information about the experimental setup can be found in [10].

## 3. Results and Discussion

### 3.1. Tensile Strength of Membrane and Structural Implications

**Figure 2:**
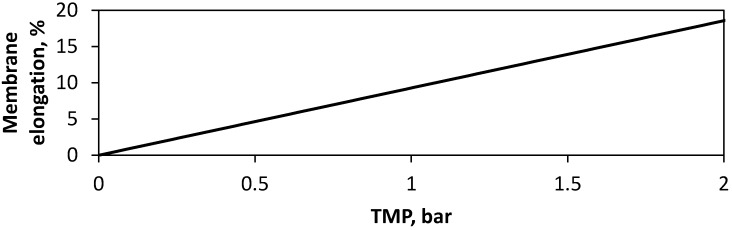
Relationship between the transmembrane pressure (TMP) applied in the Forward osmosis (FO) system and expected elongation of the membrane.

**Figure 3:**
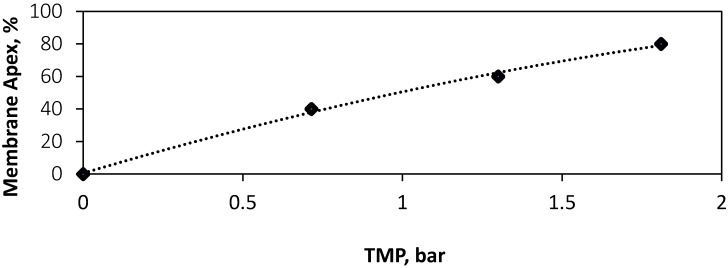
Relationship between the TMP applied in the FO system and expected apex point of the membrane as a percentage of channel height.

FO membrane samples were tensile tested to assess their Young’s modulus, to understand their physical behaviour under the pressure applied in FO processes. The results show that pressures applied in typical FO membrane process operation do not exceed the elastic region of the membrane (0–10 N), with a maximum stress of 5.82 MPa. The resulting elongation of the membrane is expected to be recoverable under low pressure. The Young’s modulus of the membrane samples was found to be 88.55 ± 0.51 MPa, showing a similar modulus than the other TFC FO membranes [32,33] and significantly lower than the equivalent TFC RO membranes which have a 500–800 MPa modulus [34,35]. This difference in modulus and therefore elasticity emphasises the need for the characterisation of subsequent effects on fluid flow within novel FO systems. As explained in Section 2.2, the Young’s Modulus can then be used in conjunction with Hook’s Law, to derive a mathematical model which describes the degree of membrane elongation expected TMP’s from 0–2 bar (Figure 2). Using the derived elongation percentage (Figure 2), with mathematical shape model (Section 2.2), a relationship expressing the membrane apex as a percentage of channel height was derived (Figure 3). This model demonstrates the expected change in channel geometry at TMP’s from 0–1.8 bar, assuming each dot spacer is a structural support, uniform deformation, and the membrane is held with exact tension. The results indicate that the channel likely undergoes an occlusion, or ‘draw channel contraction’ from 0–80% at TMP, of 0–1.8 bar, respectively. For example, the 7.7% elongation measured at 0.7 bar of pressure would lead to the membrane apex, reaching 40% of the channel height. This leads to an expected range of 0–68% during the operating TMP range of 0–1.45 bar experimentally tested our previous work [10]. These pressures and resulting occlusion at less than 2 bar (Figure 3) are known to affect the structural integrity of the membrane affect salt selectivity, leading to increased mechanical support requirements from FO draw spacers to maintain performance during pressurised conditions [20].

### 3.2. Pressure Drop Analysis

Further investigation was conducted from a fluid characterization perspective to determine a complimentary assessment of membrane behaviour in the channel during operation. A range of membrane apex percentages were simulated in ANSYS Fluent and compared with previously obtained experimental data [10] to assess the degree of occlusion likely to happen in the draw-spacer geometry during typical operation at a range of TMPs.

**Figure 4:**
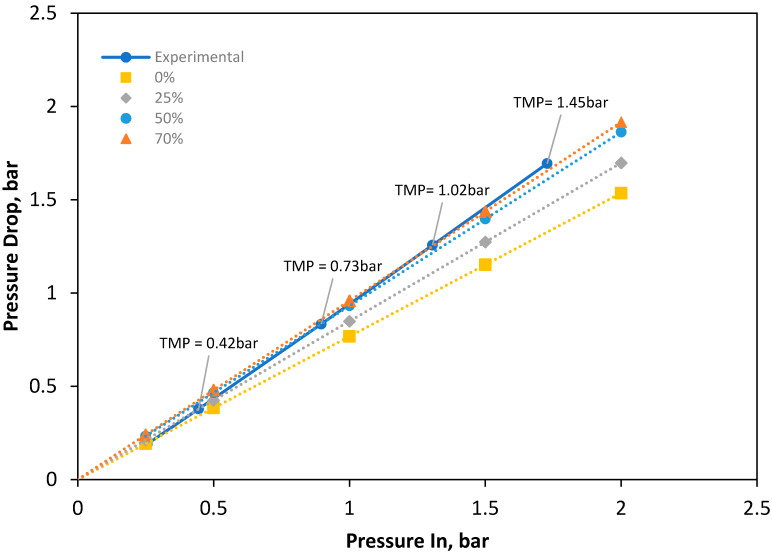
Inlet pressure plotted against pressure loss fit against experimentally collected data, ranging from 0–70% occlusion of membrane channel geometry by membrane occlusion.

Figure 4 shows pressure drops from CFD simulations of 0%, 25% and 70% occlusion, compared against experimental data at inlet pressures from 0–2 bar. The CFD data is highly linear for each level of occlusion with an R-squared value of 0.999. However, in contrast, the experimental data is not linear as would be expected and overall tends to shift between the pressure drops of different levels of occluded channels. At an inlet pressure of 0.26 bar, the experimental pressure drop most matches the clear simulated channel, with pressure drops of 0.18 and 0.19 bar, respectively. With higher inlet pressure and TMP, the experimental data shifts to more closely behaving as the 70% occluded channel (Figure 4). This non-linear trend in the experimental data, combined with how well it matches and shifts between certain levels of occlusion, indicates the nature and degree of draw channel occlusion/’contraction’. Additionally, the range of experimental data indicates that under a range of conditions the membrane tends to deform under a low TMP, with at 1 bar causing a likely 51% membrane apex of the draw channel.

### 3.3. Velocity Profile Analysis to Determine Spacer Effects on Fluid Flow

ANSYS Fluent was used to determine velocity contour profiles of interior fluid flow, at multiple simulated membrane occlusion degrees. This was performed to assess the fluid flow profile behaviour interaction with the dot spacer in the draw channel and further assess the possible effects of a membrane bending into the channel. Specifically, velocity profiles are used to assess where dead-zones and areas of high/low velocity develop in the channel [30]. All data was normalized to a consistent CFV, as opposed to flowrate due to the greater detail between occlusion degree this provides. While increasing the flowrate would demonstrate the increase in CFV in related to a thinner draw channel, information relating to velocity fluid flow profiles (i.e., low flow regions behind spacers or underneath the areas of most significant membrane deformation) is more closely related to determining current spacer/channel deficiencies.

**Figure 5:**
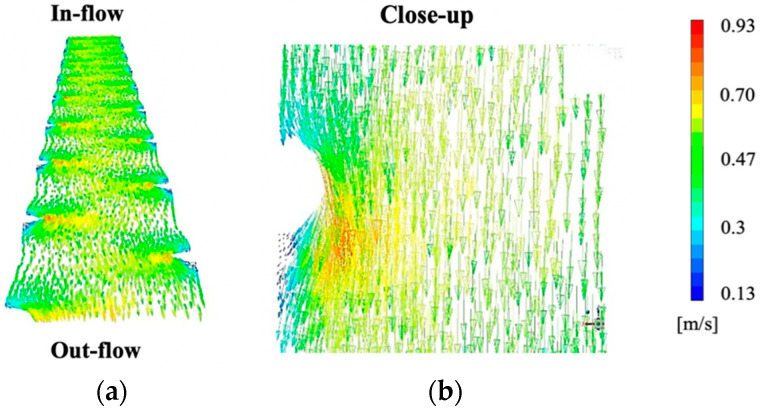
A 3D view of the 70% occluded channel simulated at 0.575 m/s demonstrating the effect of curvature on the velocity profile: (**a**) diagonal-view and (**b**) close-up of dot spacer. Simulations were performed at 0.575 m/s, as this was the highest cross-flow velocity (CFV) used in experimental testing and this most likely to give visually disparate results.

The channel velocity in Figure 5a demonstrates a cross-section view, revealing more clearly the ‘valleys’ that incline towards the supporting spacers, as well as at their lowest point medially between the spacer dots. The channel shown is at 70% occlusion, as this is the highest occlusion simulated and offers the most visually comprehensible figure, at a cross-flow velocity of 0.575 m/s. Dead zones can be seen next to the dot spacers (Figure 5b) both as an anterior cavity and more pronounced in the posterior cavity just after the spacer support. These dead zones are expected, as fluid flow behind solid supports (such as membrane spacers) is known to decrease in velocity.

From the 3D views in Figure 5, it is also clear that the velocity increases alongside the increase in membrane elevation within the channel, showing the impact of the flow, with regions of 0.32 m/s in the areas of least support and as high as 0.88 m/s in the regions of spacer support. This decrease in velocity of 64% is of particular importance when assessing the impact of the membrane occlusion in the channel, as an ideal flow profile is well-mixed with an even velocity distribution to reduce pressure loss and dead- zones.

**Figure 6:**
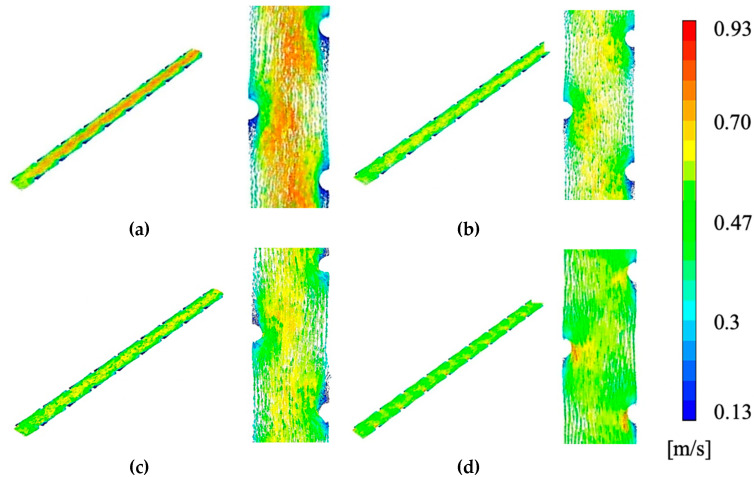
Assessment of velocity profiles with normalized inlet CFV of 0.575m/s, at (**a**) 0% occlusion, (**b**) 25%, (**c**) 50% and (**d**) 70% occlusion with close-up.

Figure 6 shows the actual flow profile with respect to the velocity profile via a vector map of the chamber geometry, at membrane apex degrees of 0%, 25%, 50% and 70%. The results were mapped in at a CFV of 0.575 m/s as this was the highest velocity used in the experimental data and subsequently most likely to simulate a vector plot with visually comprehensible differences. A 0% occlusion, as seen in Figure 6a demonstrates an almost unimpeded velocity profile, with a red-hue, ~0.7–0.9 m/s, the channel of high-speed flow down the middle of the draw chamber that indicates lower pressure drops in congruence with the lower pressure drop of the 0% apex (Figure 4). A large degree of orange and green-hue medium/high-velocity zones are found around the spacers at 0% membrane apex, indicating a large degree of fluid interaction with the spacers and thus effective spacer mixing in the draw channel, in direct contrast with the low interactions of the simulations where membrane apex reached further into the channel, as seen in Figure 6b–d. The total flow profile at 70%, seen in Figure 6d, demonstrates between the three geometries the highest degree of flow impediment as expected, with the highest degree of green-hue, ~0.35–0.4 m/s, low-speed velocity zones. Additionally, the 70% channel shows the least interaction of velocity magnitude with the dot-spacers, demonstrating a much more consistent gradient between spacers, implying a less effective spacer in terms of not only structural support but mixing and turbulence. Overall, the membrane is occluding the channel and heavily impeding the fluid flow, leading to a change in the FO membrane performance that needs further assessment.

### 3.4. Assessment of Channel Occlusion on Shear Rate

Shear strain rate analysis was performed to determine the strain rate exerted by the fluid on the membrane surface, as an indicator for the mitigation of fouling and ECP [36,37,38,39]. Complimentary to Reynolds number assessment, shear rate provides additional assessment needed when improving flux performance of FO systems [40]. Shear rate offers a more specific characterisation of the force on the membrane surface and is directly related to energy expenditure needed thus imperative to find an optimum between CP mitigation and cost.

**Figure 7:**
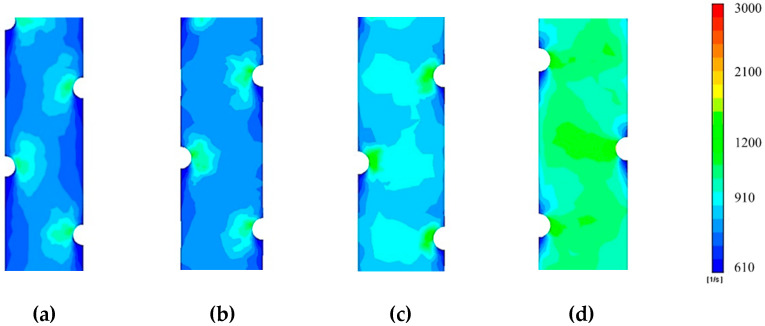
Shear rate contours of membrane surface inside PF channel geometry simulated: (**a**) total chamber contour of membrane rate at a CFV 0.10m/s and 0% occlusion, (**b**) 25% *occlusion*, (**c**) 55% occlusion and (**d**) 70% occlusion.

Figure 7 shows the flow profile with respect to shear rate via a contour map of the chamber geometry, modelled at channel occlusions between 0 and 70%. The results were mapped in ANSYS FLUENT at a CFV of 0.10 m/s, the medial velocity used in the experimental data, still able to generate a contour map with a high degree of readable gradients. Seen in Figure 7a is the total flow profile at 0%, illustrating a visually consistent shear rate contour across the entire chamber, in contrast to pressure which decreased across the channel linearly. High strain rate regions are found close to the dot-spacers, indicating that the spacer provides the highest degree of shear strain on the membrane surface when there is no occlusion considered the channel. Figure 7b–d progressively demonstrate higher strain rates, as expected due to the narrowing channel geometries. Showing the highest strain rate in (Figure 7d) is the 70% occlusion chamber, notably, the region of the highest rate at a distance furthest from all three spacer dots in the chamber, representing the point of highest membrane occlusion of the channel. This strain rate trend r multiple levels of occlusion concludes the membrane itself provides a more significant degree of the shear stress when compared to the dot spacer which is evidenced by the larger bands of shear strain between dot-spacers. A high degree of shear rate on the membrane surface is aligned with the literature with better fouling performance [36,37,38,39]. It represents an improvement in the overall ECP mitigation within the process, to be weighed against the pressure drop efficiency of the channel, which decreases with the same respective occlusion of the chamber.

The channel interior average of shear rate of the fluid was estimated using FLUENT, and simulated occlusions between 0 and 70% were chosen to represent the majority of practical operating conditions of the PF chamber geometry in line with the previous section.

**Figure 8:**
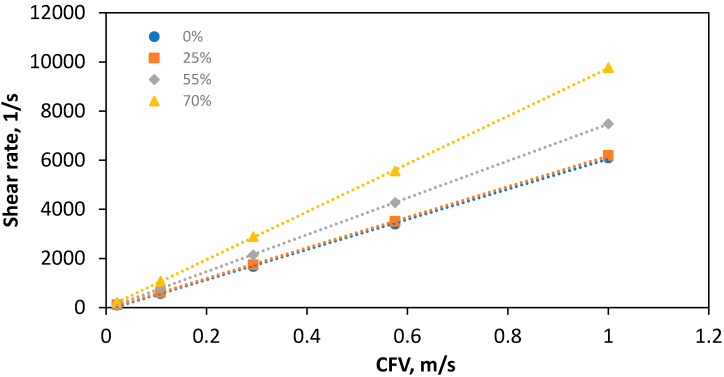
Interior average shear rate on the membrane surface between 0–70% occlusion by membrane displacement in draw channel.

Figure 8 illustrates the average shear rate across the membrane surface in the draw channels on PF membranes in operation at multiple cross-flow velocities, which were used in the original experimental tests of the module [10]. Overall, the shear rate can be seen to increase proportionally to the CFV, indicating a highly linear relationship in agreeance with the general trend found previously in [2]. At a CFV of 0.022 m/s, which was the lowest CFD condition simulated, the difference between simulated channel occlusion by the membrane is negligible, with a 54.3% difference from 0–70% occlusion, at a relatively negligible shear rate average value of 160.8. This value is in line with previously reported studies, when using a spiral wound module, but at a significantly lower velocity of 0.009 m/s [30], showing an overall trend in shear strain degree across multiple configurations. Overall for the PF draw channel used in this study, Figure 8 illustrates at low CFVs the channel occlusion can be seen to have a significant effect on membrane shear rate, implying a high difference of cake fouling [36] on the membrane regardless of the level of channel occlusion. However, at the higher CFVs, the rate values increase, yet the percentage difference between the channels actually decreases from 54.3% to 36.6%. The highest CFV of 0.575 m/s demonstrated a lower percentage difference across the levels of channel occlusion. Following this trend, at higher CFV’s the implicated fouling performance is likely less affected by membrane apex’s occluding channels.

### 3.5. Reynolds Number

An assessment of Reynolds number at the membrane surface is a useful measurement of the flow profile, a direct measurement of turbulence and an indication of mixing. Reynolds number has been previously reported in the CFD analysis of FO processes, which makes it a useful comparison point to other literature on FO processes [30]. Reynolds number provides additional insight to shear rate characterisation with a focus on mixing (turbulence) rather than a perpendicular force on the membrane surface. The Reynolds number at the membrane surface was assessed as a contour map to give an indication of flow mixing and hence the potential for ECP mitigation [41].

**Figure 9:**
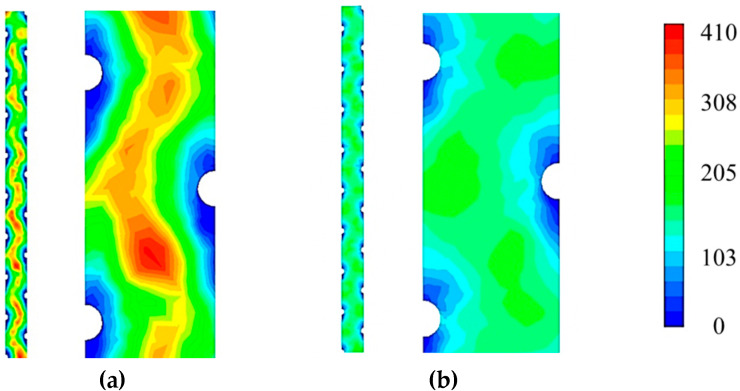
Reynolds number contours of PF channel geometry simulated at the membrane surface total chamber contour of average Reynolds Number at a CFV of 0.575m/s for (**a**) 0% and (**b**) 70% occlusion.

Flow profile as a function of Reynolds number is presented in Figure 9. A consistent flow profile was seen across the length of the chamber (Figure 9a), in contrast to the pressure along the chamber, which can be seen in the CFD and experimental data to linearly decrease (Section 3.2). This, however, is similar to the trend found with shear rate values across the length of the membrane chamber (Figure 7), showing a relationship between mixing of the flow profile and shear strain exerted on the membrane surface. Consistent with the results found from shear rate analysis (Figure 7), the areas of highest Reynolds number can be found occurring in the valleys spanning medially to the dot spacers (Figure 9b), indicating the large impact the spacers have on the mixing by producing Reynolds dead zones. A low gradient from the spacer to the middle of the channel is seen in Figure 9b, indicating that at a 70% occlusion degree the mixing occurs almost entirely at the spacers and did not increase in the middle of the channel. This decrease in mixing is due to the membrane occlusion within the channel, smoothing flow and reducing mixing and turbulence of the PF draw side. In direct contrast Figure 9b shows higher Reynolds regions (~350–400) in the centre of the channel, indicating greater spacer and mixing effectiveness. Additionally, while low in range, a Reynolds number of ~300 is an important transition point in the flow profile that influences the performance of FO separation in narrow spacer filled channels [42]. The known relationship between channel occlusion and increased overall CP supports the findings of decreased Reynolds number at the membrane surface [20]. However, the relative contributions of turbulence in the bulk flow and on the membrane surface currently leaves a research gap needing a further assessment of the bulk fluid flow.

**Figure 10:**
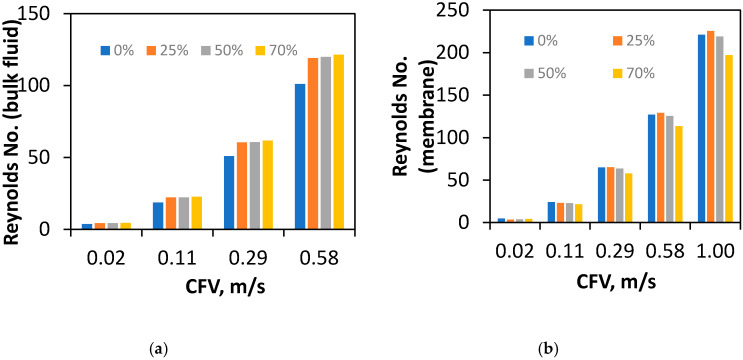
(**a**) Average Reynolds number of the interior PF channel and (**b**) average Reynolds on membrane surface.

Subsequently, a numerical average Reynolds number was calculated to determine the turbulence and mixing in the overall channel (Figure 10a). This average was calculated across the entire interior channel geometry and represents the mixing and turbulence of the bulk fluid transfer in the module to provide a complimentary assessment of the data at the membrane surface.

The Reynolds numbers of CFD simulations were calculated for channel occlusions between 0 and 70%. Similar to the analysis of shear rate in Section 3.5, this also represents a strong linear relationship. In comparison to the Reynolds number at the membrane surface, the Reynolds number of the bulk increases with a higher degree of draw-channel occlusion by the membrane. This increase indicates better mixing of the bulk fluid when draw channel contraction occurs. However, once the channel was even partially occluded at 25%, the Reynolds did not increase from the initial jump after 0% indicating the benefits are not linear. Additionally, in direct contrast to the trends of shear rate, Reynolds number does not diverge with respect to the difference between channel occlusion, showing no added mixing/ECP mitigation benefits when compared to the higher pressure loss (Section 3.2). This divergence does follow the trends set by pressure drop assessment (Section 3.3), which is to be expected as turbulence is a known source of pressure loss due to friction. However, the occluded membrane channels have a highly similar Reynolds number and provide little resolution into the effects of the membrane apex degree on spacer effectiveness.

The average Reynolds number at the membrane surface is a more specific measurement of mixing where ECP is occurring (Figure 10b). This difference shows the more significant effects of the spacers relative to the Reynolds characterisation on the membrane surface where ECP is occurring, as opposed to the general channel design. Consistent with the trend of decreasing Reynolds number over occlusion (Figure 9), the membrane surface average decreases by 10.9%. The surface decrease average is in direct contrast to the bulk flow average Reynolds number and underlines the tradeoff between bulk Reynolds and a wall average on the membrane surface, needing further work on the relative contribution of both factors to ECP.

## 4. Conclusions

The effects of membrane draw channel contraction were characterised using experimental and CFD data to provide insight into FO draw channel hydrodynamics. The pressure loss of multiple simulated membrane occlusions in the draw-side chamber was matched against experimental data to determine the level of membrane displacement found operating in the FO process. From tensile data, at typical FO TMPs, the membrane was found to stretch into the draw channel and occlude the fluid flow 0–67.7% of the membrane channel height, when TMP of 0–1.45 bar was applied (respectively). Fluid velocity was found to be heavily impeded by the membrane occluding the channel and blocking fluid flow, showing significant overall impact by the membrane on the channel hydrodynamics. Shear rate along the membrane surface and inside the channel was found to improve significantly (89.9%) and would indicate better CP mitigation and better fouling performance. However, the span over which the difference between 0 and 70% occlusion ranges decreases with higher CFV showing smaller improvement at increasing CFV’s between occlusion. This decrease in effectiveness at mitigating CP is a trade-off that needs to be balanced at the inherently higher CFV’s of a narrowing channel. Reynolds number of the interior was found to increase as a bulk average within the channel (overall + 20.2%) but decrease on the membrane surface (−10.9%). The implications of the difference in trends between turbulence near the membrane surface, and bulk flow demonstrate the need to determine the relative contribution of both factors, for the further development of optimal spacers in FO processes that involve feed pressure.

The velocity, shear and Reynolds assessment all demonstrate the trade-off needed to be made when further designing spacers for FO draw-channels, and furthermore, the additional required work on characterising the relative contribution of both factors to ECP and FO performance. Overall, outcomes of this study show a more accurate assessment of the level of draw channel occlusion than current literature, as well as an in-depth assessment of hydrodynamic effects within the draw-channel that impact FO membrane performance.
